# A case report of atypical hemiplegic migraine with nonheadache onset in a Chinese child

**DOI:** 10.1186/s12883-021-02302-9

**Published:** 2021-07-06

**Authors:** Hui Chen, Xiaolan Sun, Ruiyan Wang, Zhaoshi Yi, Zhixin Huang, Jihua Xie, Xiongying Yu, Yong Chen, Jianmin Zhong

**Affiliations:** Department of Neurology, Children’s Hospital of Jiangxi Province, Nanchang, 330006 China

**Keywords:** ATP1A2, Alternating hemiplegia, Hemiplegic migraine, Case report

## Abstract

**Background:**

Hemiplegic migraine (HM) is an uncommon subtype of migraine with aura including motor weakness. The core symptoms of HM are headache and motor weakness. However, we report a rare case of atypical HM with nonheadache onset in a Chinese child who was misdiagnosed several times.

**Case presentation:**

We report a Chinese boy whose onset was sudden when he was 3 years old. He presented with a variety of phenotypes, including fever, vomiting, alternating hemiplegia, and drowsiness, but no headache in the initial stages. Magnetic resonance imaging (MRI) demonstrated unilateral cerebral oedema during the initial episode of hemiplegia. These symptoms recurred many times. As the disease progressed, the patient developed episodic headache. The patient was misdiagnosed several times with encephalitis, alternating hemiplegia of childhood (AHC) and mitochondrial encephalopathy. Whole-exome next-generation sequencing revealed a de novo heterozygous missense mutation in the ATP1A2 gene(p.Gly715Arg) classified as pathogenic and eventually led to a diagnosis of HM when he was 11 years old. Flunarizine was subsequently administered, and no recurrence was found during follow-up.

**Conclusions:**

HM in children may be atypical in the initial stage of the disease, which could manifest as fever, alternating hemiplegia and drowsiness but no headache at the onset. This could easily lead to misdiagnosis. With age, it may eventually manifest as typical HM. Therefore, attention should be given to differentiation in clinical practice.When similar clinical cases appear, gene detection is particularly important, which is conducive to early diagnosis and treatment.

## Background

Hemiplegic migraine (HM) is an uncommon subtype of migraine with aura including motor weakness. The onset is generally in adolescence between 12 and 17 years, and the overall estimated prevalence is 0.01% [1–3]. HM can be divided into familial hemiplegic migraine (FHM) and sporadic hemiplegic migraine (SHM). FHM is defined by the usual symptoms of HM, with at least one first- or second-degree relative of the patient also having migraine with aura including motor weakness [2]. Specific genetic subforms have been identified. There are mutations in the CACNA1A gene (coding for a calcium channel) on chromosome 19 in FHM1, while FHM2 and FHM3 have mutations in the ATP1A2 gene (coding for a K/Na-ATPase) on chromosome 1 and the SCN1A gene (coding for a sodium channel) on chromosome 2, respectively [2, 4].

The core symptoms of HM are headache and motor weakness. We report a case of atypical hemiplegic migraine with nonheadache onset in a Chinese child when he was 3 years old. Since there was no headache in the early stages of the disease, he was misdiagnosed several times. This was a rare case, and we are therefore reporting it to deepen the understanding of this disease and reduce the occurrence of misdiagnosis.

## Case presentation

The patient’s birth history, family history, and developmental history prior to onset were normal. The first episode occurred at 3 years old. The boy was admitted to the Children’s Hospital of Jiangxi Province with fever (up to 39℃), vomiting, drowsiness, and right hemiplegia that did not improve during sleep for 2 days. If his body was in pain, he could usually articulate the sensation of pain, but the child had no headache attack. Head magnetic resonance imaging (MRI) after admission showed low signal on T1-weighted imaging (T1WI), high signal on T2-weighted imaging (T2WI), and high signal on diffusion weighted imaging (DWI) in the left parieto-occipital cortex. In addition, a septum pellucidum cyst was seen (this result was only shown in the MRI report, but unfortunately, the original picture was lost over time). The white blood cell count and protein, sugar and chloride levels in the CSF were normal. Blood lactic acid and plasma ammonia were 1.4 mmol/L (normal range 0.5–1.6 mmol/L) and 43 µmol/L (normal range 18–72 µmol/L), respectively. The patient was diagnosed with encephalitis and was given acyclovir against virus and mannitol to reduce intracranial pressure. The symptoms of vomiting disappeared on the second day after admission. His body temperature was normal on the third day after admission, and his limb movements returned to normal on the sixth day after admission.

He experienced a second attack 2 months after the first. The patient was admitted with fever (up to 38℃), vomiting, and left hemiplegia for 12 h. During this episode, the child still did not complain of headache. Head MRI after admission showed low signal on T1WI, high signal on T2WI, and high signal on DWI in the right parieto-occipital cortex. The patient was given acyclovir against virus and mannitol to reduce intracranial pressure. All symptoms (fever, vomiting, hemiplegia) returned to normal on the third day after admission.

When the patient was 4 years old, the third episode occurred. On admission, in addition to fever, vomiting, and left hemiplegia for 1 day, there were four convulsions (generalized tonic–clonic seizures, each convulsion lasted approximately 1–3 min) on the same day, accompanied by aphasia and drowsiness. With the improvement of aphasia symptoms, the child began to have headache. The location and character of the headache were not clearly described, and the headache lasted for approximately 3 h before relief. Head MRI after admission showed low signal on T1WI, high signal on T2WI, and high signal on DWI in the right parieto-occipital cortex. The electroencephalogram(EEG) showed diffuse slow waves and no epileptic discharge. The white blood cell count and protein, sugar and chloride levels in the CSF were normal. The lactic acid and ammonia levels of CSF were 1.4 mmol/L (0.7–2.1 mmol/L) and 44 µmol/L (10–45 µmol/L), respectively. Blood lactic acid and plasma ammonia were 1.6 mmol/L (0.5–1.6 mmol/L) and 86 µmol/L (18–72 µmol/L), respectively. Alternating hemiplegia of childhood (AHC) and mitochondrial encephalopathy were suspected at the time. The patient was given mannitol to reduce intracranial pressure and received supplementation with vitamin B1, vitamin B6, coenzyme Q, and levocarnitine. All symptoms returned to normal on the sixth day after admission.

**Fig. 1:**
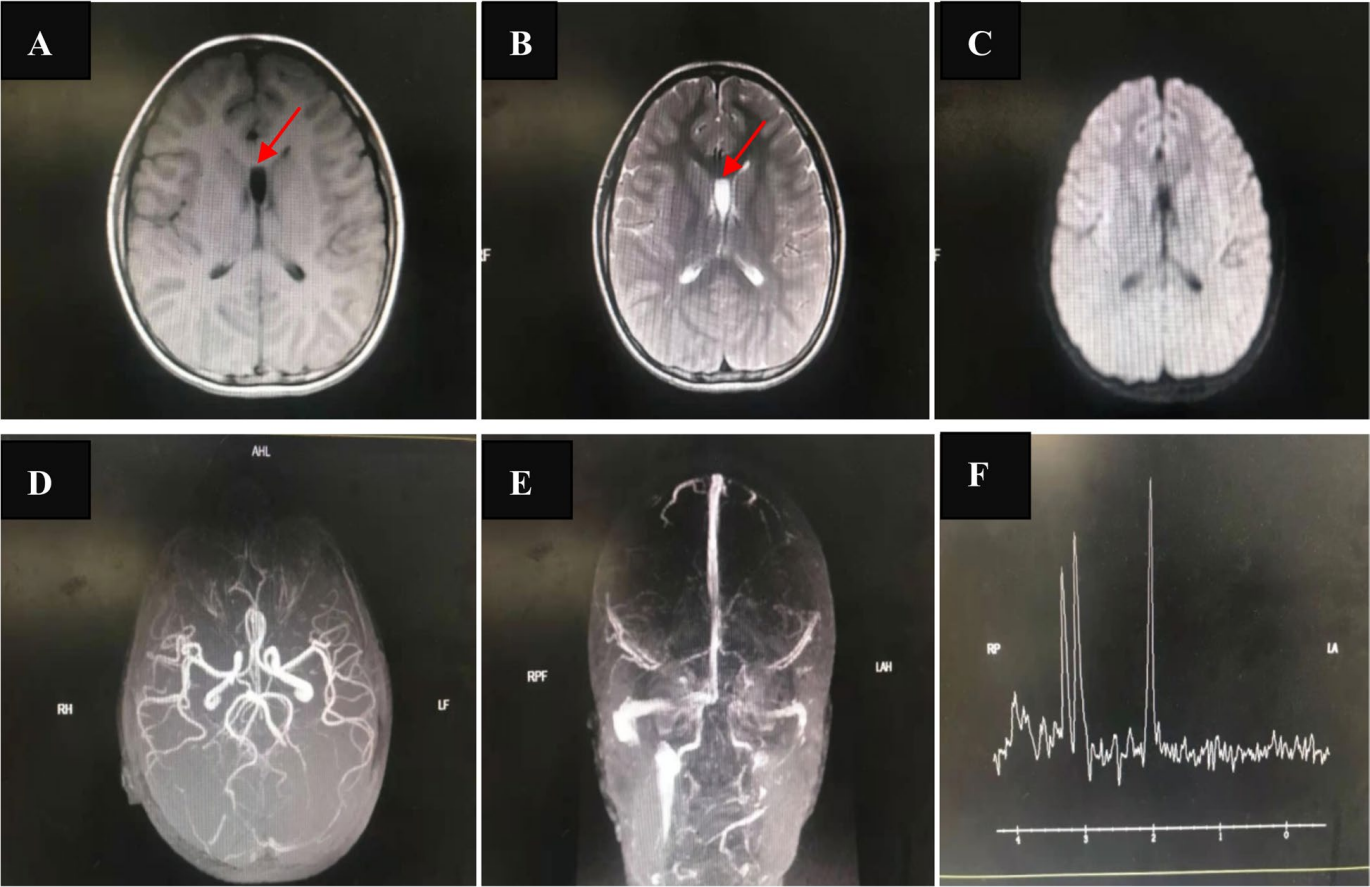
MRI of the brain during the last episode. There were no abnormal signals except the preexisting septum pellucidum cyst (The arrow) which was similar to that before. **A** T1WI; **B** T2WI; **C** DWI; **D** Magnetic resonance angiograghy (MRA); **E** Magnetic resonance venography (MRV); **F** Magnetic resonance spectroscopy (MRS)

The fourth attack occurred at the age of 11. Two months before admission, headache occurred, mainly on one side of the front head, with no blurred vision. After approximately 10 min, headache was relieved by itself. However, the headaches became more intense, taking up to 2–3 h to relieve 1 week before admission. One day before admission, fever, vomiting, left hemiplegia, aphasia, and drowsiness symptoms appeared again. However, as the symptoms of aphasia improved, the headache became more intense. The MRI showed no abnormal signals except for the persistent septum pellucidum cyst (Fig. 1). Ultrasound of the heart, bilateral internal carotid artery, external carotid artery and common carotid artery showed no abnormalities. As before, these symptoms returned to normal after mannitol and multivitamin supplementation. However, as the disease progressed, the patient developed cognitive impairment.

**Fig. 2:**
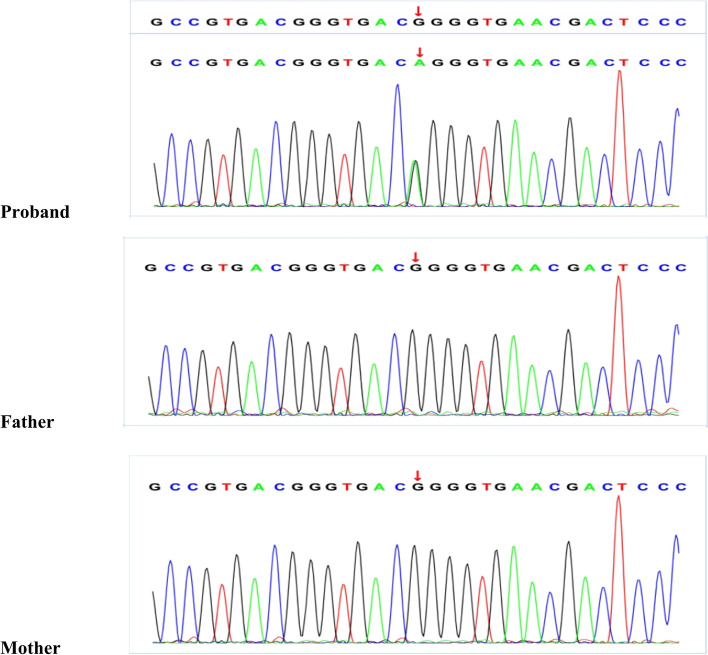
A denovo heterozygous missense mutation in the ATP1A2 gene: c.2143(exon16)G > A (p.Gly715Arg) (NM_000702) in the proband, and the mutation was not found in the proband’s parents

To further clarify the diagnosis, we conducted mitochondrial DNA sequencing analysis and found no mutation sites with high pathogenicity in mitochondrial DNA. Whole-exome next-generation sequencing (NGS) of the nuclear genome revealed a de novo heterozygous missense mutation (Fig. 2) in the ATP1A2 gene (p.Gly715Arg) classified as pathogenic (PS1 + PS2 + PM1 + PM2 + PP3) that eventually led to a diagnosis of SHM when he was 11 years old [5]. Flunarizine (5 mg, oral, 1 time per day before bed) was subsequently administered, and it has now lasted for 4 months without any adverse effects. No recurrence was found during follow-up.

## Discussion and conclusions

This case presented with a variety of phenotypes that included fever, vomiting, alternating hemiplegia, convulsions, drowsiness, aphasia, episodic headache, unilateral cerebral oedema and cognitive impairment. HM caused by mutations in the ATP1A2 gene has been reported [6, 7]. However, it is rare to report so many symptoms that are recurrent and varied in a single child. In addition, the onset of the HM was atypical because no headache apeared at the onset. He was misdiagnosed many times.

In the early stages of the disease, fever, vomiting, drowsiness, and hemiplegia were observed. A head MRI showed unilateral parieto-occipital cortical oedema. The white blood cell count and protein, sugar and chloride levels in the CSF were normal. These manifestations were consistent with the diagnosis of encephalitis [8]. However, the patient subsequently had several similar episodes, and we believed that this patient might not be consistent with the diagnosis of encephalitis. At this time, because the patient presented with fever, vomiting, drowsiness, hemiplegia and convulsions, MRI showed unilateral cortical oedema, which occurred repeatedly. We considered that the child might suffer from mitochondrial encephalopathy induced by infection. However, the patient’s blood or cerebrospinal fluid lactic acid levels were normal many times, and there was no similar family history. Subsequent mitochondrial DNA sequencing analysis was normal. In general, the clinical data and related examinations did not meet the diagnostic criteria of mitochondrial encephalopathy.

Many of the patient’s clinical features supported the diagnosis of AHC, such as alternating hemiplegia, developmental retardation, and convulsions. In addition, NGS of the nuclear genome revealed a de novo heterozygous missense mutation in the ATP1A2 gene (p.Gly715Arg) classified as pathogenic. Mutations in the ATP1A2 gene can cause hemiplegic migraine type 2, common migraine, and basilar migraine but can also cause a very small number of AHC cases [9–14]. However, our patient did not experience improvement of hemiplegia during sleep, as described in previous reports [15, 16]. In addition, studies have shown that brain MRI of patients with AHC always shows normal or nonspecific findings [15, 16]. However, the brain MRI of our patient showed unilateral cortical oedema. In conclusion, the basis for the diagnosis of AHC in the child was still insufficient.

With the progression of the disease, the child developed paroxysmal headache, accompanied by hemiplegia and aphasia. Symptoms were recurrent and mostly reversible. First- or second-degree relatives had no migraine with aura including motor weakness. These core symptoms and characteristics were consistent with the diagnostic criteria of SHM [2]. It has been widely reported that HM can sometimes be accompanied by symptoms such as fever, convulsion, altered consciousness (drowsiness or even coma), and cognitive impairment, as well as cerebral oedema seen in head MRI [3, 6, 7]. All of these symptoms occurred in our patient. NGS of the nuclear genome revealed a de novo heterozygous missense mutationin the ATP1A2 gene (p.Gly715Arg) classified as pathogenic and eventually helped him to be diagnosed with SHM when he was 11 years old. The duration of symptoms is usually 20–60 min. In some cases, the aura and hemiplegia may onset quickly and simulate an ischaemic attack [17]. Complete recovery from attacks is the rule, but in severe HM, hemiplegia and altered consciousness may persist for weeks until normal [3]. In this case, symptoms lasted from 3 days to 1 week until total recovery. The child had recurrent headache attacks for approximately 2 months before the fourth attack, and headaches became more intense 1 week before admission. As the child grew older, the headache increasingly affected his life. In addition to this, he only had four episodes. However, as the disease progressed, the patient developed cognitive impairment, and cognitive decline was a serious problem. Consequently, flunarizine was subsequently administered, and it has now lasted for 4 months without any adverse effects. No recurrence was found during follow-up. However, the exact length of treatment is still unclear because this is an atypical case, and follow-up of his symptoms and regular monitoring of the side effects of flunarizine will help determine this. In the case report, we found a significant phenomenon. In addition to the evolution of symptoms, the child’s MRI evolved from cerebral oedema at the beginning of the disease to normal MRI at the last attack. However, the picture of the MRI has been lost. This is the limitation of this case report, but it did not affect the conclusions because we knew the MRI findings from the MRI report provided by the guardian. In addition, when the third episode occurred, we performed an EEG on the child when they had hemiplegia but did not complain of headache. The EEG showed diffuse slow waves and no epileptic discharge. However, it was regrettable that we did not perform long-term EEG in this child to understand the ictal and interictal EEG manifestations. This is conducive to our recognition of HM [18]. At present, the pathogenesis of migraine is still unknown and focuses mainly on neurophysiology, especially cortical brain disorders [19–21]. This needs further study in the future.

In summary, HM in children may be atypical in the initial stage of the disease, which could manifest as fever, alternating hemiplegia, and drowsiness but no headache at the onset. This could easily lead to misdiagnosis. With age, it may eventually manifest as typical HM. Symptoms were recurrent and variable. Therefore, attention should be given to differentiation in clinical practice. When similar clinical cases appear, gene detection is particularly important, which is conducive to early diagnosis and treatment.

## Data Availability

The datasets used and/or analysed during the current study are available from the corresponding author on reasonable request.

## Abbreviations

HM: Hemiplegic migraine
FHM: Familial hemiplegic migraine
SHM: Sporadic hemiplegic migraine
MRI: Magnetic resonance imaging
T1WI: T1-weighted imaging
T2WI: T2-weighted imaging
DWI: Diffusion weighted imaging
AHC: Alternating hemiplegia of childhood
MRA: Magnetic resonance angiograghy
MRV: Magnetic resonance venography
MRS: Magnetic resonance spectroscopy
NGS: Next generation sequencing
